# Misdiagnosed Spontaneous Carotid Cavernous Sinus Fistula

**DOI:** 10.5811/cpcem.2019.4.42247

**Published:** 2019-05-29

**Authors:** Maureen Canellas, Navneet Cheema

**Affiliations:** University of Chicago, Department of Emergency Medicine, Chicago, Illinois

## Abstract

A 63-year-old female presented to the emergency department with worsening left-sided blurry vision and diplopia. She had previously seen several physicians and had been diagnosed with common ocular conditions – keratoconus and dry eye. However, despite treatment her symptoms were worsening. By the time her true underlying diagnosis was treated, she was left with permanent vision loss. This case report discusses the presentation, diagnosis, and treatment of her rare condition.

## INTRODUCTION

A carotid cavernous fistula (CCF) is a rare connection between the carotid artery and cavernous system. It can occur spontaneously or traumatically.^1^ Spontaneous CCFs are more commonly insidious and misdiagnosed.^2^ They frequently present with ophthalmoplegia and changes in vision.^3^ When left untreated, the damage to cranial nerves and vision loss can become permanent.^2^ In this case, we present the diagnosis, treatment, and outcome of a patient with a spontaneous CCF that had been misdiagnosed for months.

## CASE REPORT

**Image 1:**
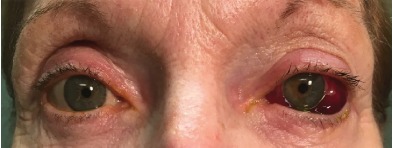
Physical exam revealing a large, left-sided subconjunctival hemorrhage, proptosis, and chemosis. Patient consent was obtained to use this Image.

A 63-year-old female without past medical history presented with left eye pain and headaches for five months. These symptoms acutely worsened over the prior three days with associated blurry vision and diplopia. The vision changes started gradually and without preceding trauma or an inciting event. They were associated with an intermittent left ear whooshing sound. Over the previous five months, she had one primary care visit, two neurology visits, five emergency department (ED) visits, and seven ophthalmology visits for these symptoms. At her previous visits, she was diagnosed with dry eye and keratoconus, an abnormal bulging of the cornea leading to vision changes, eye redness and pain, and headaches. In the ED her eye exam revealed a left large subconjunctival hemorrhage and chemosis (Image 1).

**Image 2:**
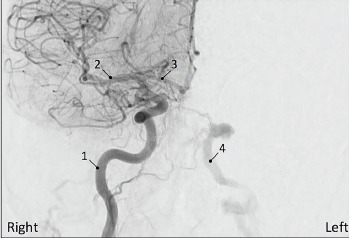
Right carotid system angiogram revealing cross-filling into the left carotid system and left inferior petrosal sinus fistula. (1) Right internal carotid artery; (2) middle cerebral artery; (3) anterior cerebral artery; (4) left internal carotid artery.

**Image 3:**
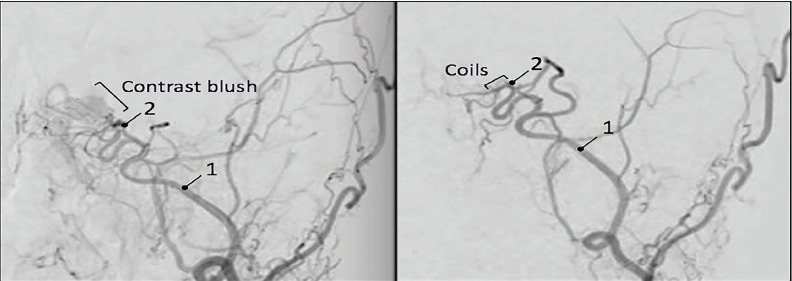
(Left) Internal carotid artery angiogram with blush at the site of the fistula, and (right) subsequent closure post-coiling. (1) Cavernous segment of left internal carotid artery; (2) ophthalmologic segment of left internal carotid artery.

Her neurologic exam revealed partial left cranial nerve III, IV, and VI palsies. Laboratory testing and computed tomography (CT) of the head were inconclusive. During the previous five months of visits, she had negative imaging including two CTs of her head without contrast, magnetic resonance imaging (MRI) of her brain with and without contrast, MR angiogram and venogram of her brain, MRI of her orbits with and without contrast, and an ophthalmic ultrasound of her left eye. The emergency team contacted the patient’s ophthalmologist given her worsening symptoms despite negative imaging. Her ophthalmologist reported a concern about an ongoing CCF despite continued negative imaging and recommended consulting neurosurgery. Given her cranial nerve deficits and acute worsening of symptoms, the neurosurgery team immediately consented and prepped the patient for diagnostic and therapeutic angiography. Subsequent digital subtraction angiogram was performed with direct localization of the fistula between the internal carotid artery and the cavernous sinus (Image 2). The fistula was coiled with complete closure of the fistula (Image 3).

Overall, this patient had a four-month delay between initial symptoms and definitive treatment. At the time of discharge, her cranial nerve III and VI palsies resolved and cranial nerve IV palsy partially improved; however, her diplopia and blurry vision remained. These symptoms remained at the patient’s one-year follow-up.

## DISCUSSION

This patient was suffering from a rare spontaneous CCF. It is an uncommon condition that can easily be misdiagnosed as conjunctivitis or common ocular problems. CCF is important for the emergency physician to recognize given its potential for morbidity. All CCFs can be classified as direct or dural. Direct fistulas, denoted as high flow, connect the cavernous sinus directly with the intracavernous carotid artery. Dural fistulas, denoted as low flow, connect the cavernous sinus with a branch of the intracavernous carotid artery. Our patient’s angiogram findings indicated a dural CCF. Direct fistulas are commonly due to traumatic or spontaneous intimal tears in the artery, while dural fistulas are idiopathic and theorized to be related to genetic predisposition or hypertension.^1^,^4^,^5^

Both forms present with primarily ocular and neurologic signs and symptoms, ranging from subjective or ocular bruit (80%), blurry vision (32%), headache (84%), diplopia (88%), proptosis (72%), chemosis (55%), and conjunctival injection (44%).^3^,^6^ In terms of ophthalmoplegia, cranial nerve VI is the most common palsy, followed by cranial nerves III and IV.^3^ When a fistula is suspected, advanced imaging such as CT, CT angiography or MRI of the head is required for diagnosis. These images may only show proptosis and expansion of the cavernous sinus and ophthalmic drainage systems and do not reliably localize the fistula.^7^ Thus, digital subtraction angiography remains the gold standard for diagnosis. This modality allows for simultaneous treatment via embolization.

**CPC-EM Capsule**

What do we already know about this clinical entity?

A carotid cavernous fistula (CCF) is a rare connection between the carotid artery and cavernous system that can occur spontaneously or post trauma.

What makes this presentation of disease reportable?

Spontaneous CCFs are not frequently described in the literature and have higher rates of morbidity due to their insidious nature.

What is the major learning point?

Emergency physicians should be aware of the diagnosis of CCF as it can masquerade as non-emergent ocular conditions, resulting in delayed diagnosis and vision loss.

How might this improve emergency medicine practice?

This case is an important reminder to consider CCF as a cause of spontaneous monocular ophthalmoplegia or vision changes in patients with negative imaging.

A minority but not insignificant number of patients with direct fistulas sustain intracranial bleeds and life-threatening epistaxis when left untreated.^2^,^6^ However, rates of permanent ophthalmoplegia and vision loss are lower than that of dural fistulas.^8^ The high-flow nature of the fistula can lead to the continued tearing of intracerebral vessels, but also allows for an acute onset of symptoms leading to prompt diagnosis and early definitive management.^3^ Since low-flow fistulas have a more subacute presentation, they are commonly misdiagnosed as conjunctivitis or other common ocular conditions by multiple providers prior to appropriate management. This delay in treatment correlates to a 20–30% prevalence of permanent vision loss for dural fistulas.^2^

## CONCLUSION

While direct CCFs present clearly, dural CCFs commonly masquerade as a non-emergent ocular condition. This patient had a several-month delay in diagnosis and definitive treatment, leading to permanent vision loss. Emergency physicians should not underestimate this condition’s noteworthy, sight-robbing potential.
